# Bayesian predictive model of Ebola fatality: Tenth Ebola epidemic in the Democratic Republic of the Congo

**DOI:** 10.4102/jphia.v16i4.1533

**Published:** 2025-12-12

**Authors:** John Kamwina Kebela, Prince Kimpanga, Jean Nyandwe Kyloka, Godefroid Musema, Rostin Mabela, Radjabu Bigrimana, Olivier Mangapi, Berthe Barhayiga, Etienne Bwira Mwokozi, Simon Ntumba, Jack Kokolomami, Sylvain Munyanga Mukongo

**Affiliations:** 1 School of Public Health, Faculty of Medicine, University of Kinshasa, Kinshasa, Democratic Republic of the Congo; 2 African Centers for Disease Control and Prevention, Addis Ababa, Ethiopia; 3 Department of Mathematics and Computer Science, Faculty of Science, University of Kinshasa, Kinshasa, Democratic Republic of the Congo; 4 Department of Educational and Vocational Guidance, Ilebo Higher Pedagogical Institute, Ilebo, Democratic Republic of the Congo; 5 Department of Anaesthesia and Intensive Care, Faculty of Medicine, University of Kinshasa, Kinshasa, Democratic Republic of the Congo

**Keywords:** validation, internal, subjective Bayes model, fatality, Ebola

## Abstract

**Background:**

This study aimed to identify the clinical signs and symptoms most associated with fatal outcomes in Ebola virus disease (EVD) using a Bayesian framework.

**Aim:**

The goal was to develop a prognostic model capable of predicting mortality in EVD patients treated in Ebola Treatment Centres (ETCs) based on observed clinical indicators.

**Setting:**

A retrospective expert-based study of the 10th Ebola outbreak was conducted to identify key mortality factors using hypothetical cases in the Democratic Republic of the Congo.

**Methods:**

Clinical experts assessed mortality predictors in Ebola cases using Bayesian methods to estimate likelihood ratios and post-test probabilities, with analyses conducted in Excel and SPSS.

**Results:**

Eight clinical factors were identified as potential predictors of poor outcomes in Ebola virus disease. Five showed strong associations with mortality: deterioration in general condition and comorbidity, hemorrhagic syndrome, neurological disorders, biological deterioration with dehydration, and high viral load at diagnosis. Internal validation using 42 hypothetical cases demonstrated excellent performance (sensitivity [*Se*] = 97.4%, specificity [*Sp*] = 100.0%, positive predictive value [PPV] = 100.0%, negative predictive value [NPV] = 75.0%, accuracy = 97.6%) and strong expert agreement (κ = 0.84).

**Conclusion:**

The model demonstrated strong internal validity in predicting mortality from Ebola virus disease. Among five key predictors, bleeding syndrome, neurological disorders, and biological alteration with dehydration were the most accurate, each correctly predicting fatal outcomes in 83% of cases.

**Contribution:**

This Bayesian model offers a useful decision-support tool for managing Ebola outbreaks.

## Introduction

The Ebola virus (EBOV) causes acute and severe disease. This virus was first identified in 1976 during two simultaneous outbreaks in South Sudan and the Democratic Republic of the Congo (DRC).^1^ Ebola virus is very often fatal in humans, with high case fatality rates ranging from 25% to 90%.^1^ However, as of July 2015, 27 741 cases of Ebola virus disease (EVD) and *11 284 deaths* were reported in West Africa, particularly in Guinea, Liberia and Sierra Leone.^2^ In 2014, a total of 162 (7.3%) individuals with confirmed or probable EVD among 2210 health workers with more or less than 15 years of professional experience died during the epidemic in Guinea.^3^

The tenth EVD epidemic in the DRC (in the provinces of North Kivu and Ituri) was the second most deadly epidemic (2280 deaths [case fatality rate of 66%]) after the epidemic in West Africa in 2014 (2196 deaths and 1075 cured patients reported by December 2019).^4,5^

Since the emergence of EVD, its case fatality rate has remained alarmingly high. Therefore, identifying signs or symptoms that worsen a patient’s condition and may lead to a fatal outcome is essential for improving clinical management. In this study, we aim to determine which clinical characteristics observable in Ebola Treatment Centres (ETCs) (signs, symptoms and biological variables) can effectively predict fatal outcomes in patients with EVD. Early detection of aggravating factors is crucial for optimising patient care in these settings. To address this research question, we will employ a Bayesian approach to identify predictors of mortality.

Mark J. Siedner and Lawrence highlighted that large-scale threats posed by EVD mainly occur in countries with weak public health systems^6^; the DRC is no exception; the country consistently records high case fatality rates during every Ebola outbreak (see Table 1)^7^.

**TABLE 1:** Chronology of Ebola virus disease epidemics and the number of deaths in the Democratic Republic of the Congo from 1976 to 2022.

Order	Year	Dates	Main outbreak areas	Strain	Deaths	Mortality rate (%)	Duration (days)
1st	1976	August–November	Yambuku, Equateur	EBOV	280	88	-
2nd	1977	May–June	Tandala, Equateur	EBOV	1	100	-
3rd	1995	May–June	Kikwit, Bandundu, Kwuilu	EBOV	250	79	-
4th	2007	August–November	Mweka, Kasai-Occidental/Central	EBOV	187	71	-
5th	2009	December 2008 – February	Mweka, Kaluamba, Kasai-Occidental	EBOV	15	47	-
6th	2012	June–November	Isiro, Oriental Province	EBOV	36	47	-
7th	2014	August–November	Djera, Boende, Tshuapa	EBOV	49	74	-
8th	2015	May–July	Likati, Orientale, Bas Uélé	EBOV	4	50	-
9th	2018	08 May – 24 July	Wangata, Bikoro, Equateur	EBOV	33	61	78
10th	2020	01 August and 18–25 June	North‒South, South Kivu and Ituri	EBOV	2280	66	695
11th	2020	01 June – 18 November	Mbandaka, Equateur	EBOV	55	42	172
12th	2021	07 February – 03 May	Biena, Butembo, North Kivu	EBOV	6	50	86
13th	2021	08 October – 16 October	Beni, North Kivu	EBOV	9	82	100
14th	2022	23 April – 04 July	Mbandaka, Equateur	EBOV	5	100	73
15th	2022	21 August – 27 October	Beni, North Kivu	EBOV	1	100	53

*Source:* Judson SD, Munster VJ. The multiple origins of Ebola disease outbreaks. J Infect Dis. 2023;228(Suppl 7):S465–S473. https://doi.org/10.1093/infdis/jiad352

EBVO, Ebola virus.

Numerous researchers have conducted investigations to identify risk factors that may worsen the health status of patients affected by Ebola.^8,9,10,11,12,13,14,15,16,17,18^ These studies uniformly employed frequentist statistical methods and were limited to data derived from a single source, namely, one ETC, thus preventing the integration of new data from other treatment sites or from subsequent outbreaks. In contrast, a Bayesian approach offers notable advantages: it is more flexible, allows the incorporation of prior knowledge and facilitates better control of errors, particularly in the context of clinical research. By enabling the continual updating of predictive models as new data become available, Bayesian methods are particularly suited for responding to emerging threats like Ebola epidemics.

At present, Bayesian methods hold a prominent position in specialised biostatistics publications, and their adoption is gradually expanding within the epidemiological literature.^19^

The outcomes of patients with EVD are uncertain; therefore, the Bayesian approach is ideal in this context because it integrates the subjective and objective aspects of the phenomena to be studied. Table 1 shows the mortality of EVD from the first case to date in the DRC.

## Research methods and design

### Study design

This was a retrospective cross-sectional study in which the opinions of Ebola experts on Ebola management in the ETC were collected during the tenth epidemic.

The experts in EVD management listed 50 factors that affect the severity of EVD. Through consensus, they grouped the various factors cited into eight key independent factors influencing EVD mortality. The elements, grouped into exclusively independent factors, are shown in Table 2.

**TABLE 2:** Factors and symptoms and/or clinical signs of the severity of Ebola virus disease according to Ebola management experts in the Democratic Republic of the Congo.

Number	Factors (signs/symptoms) and/or patient dying of Ebola	Grouping of factors (into independent and mutually exclusive elements)	Operational definition
1	Persistent fever	Fever over 38 °C Uncontrolled fever	Any person with a body temperature > 37 °C
2	Alteration of general condition and comorbidity	Physical asthenia General fatigue State of prostration Altered general condition Malnutrition Late arrival Hypertension Dyspnoea Not vaccinated against Ebola Medical history Age under 5 years and/or over 45 years Rash	Any person with an altered general condition Any person with a major morbidity in their medical history
3	Bleeding syndrome	Bleeding and altered consciousness Redness of the eyes External haemorrhage Haematemesis Melena Haemorrhage Permanent bleeding Anaemia Decompensated anaemia	Any person presenting with a set of signs and symptoms and biological manifestations related to bleeding at a particular site
4	Pain syndrome	Joint pain Chest pain Muscle pain Sore throat Headache Myalgia Arthritis	Any patient reporting localised or generalised pain
5	Digestive signs	Diarrhoea Vomiting Anorexia Abdominal problems Abdominal pain Hiccups	Any patient with at least one sign of digestive system damage
6	Neurological disorders	Coma Unconsciousness Psychosis Convulsions Neurological disorders Aggression Loss of vision	All signs and symptoms indicating damage to the nervous system
7	Biological alteration and dehydration	Jaundice Impaired liver balance Biological state disturbed between Dx and follow-up (haematological factors, biochemical factors, protein factors, etc.) Hyperglycaemia Impaired renal function Dehydration	Any biological alteration noted in the laboratory and reported
8	High viral load and/or detection threshold at the time of diagnosis	High viral load and/or detection threshold at diagnosis	Any sample with a positive test result in which the viral specimen has been quantified by a quantitative method

In this study, the inclusion criteria were applied to all Ebola management experts who agreed to be available throughout the study period and who had worked at an ETC in West Africa between 2014 and 2015 and/or in the DRC between 2018 and 2020.

### Data collection

We used a paper questionnaire to collect data on the opinions of the Ebola management experts.

### Data entry

The experts’ opinions on the 42 hypothetical patient cases in the ETC were entered into Microsoft^®^ Excel. Consensus data were also entered using the same software.

### Data analysis

Microsoft Excel was used to programme all the Bayes mathematical modelling formulae, calculate kappa statistics to measure the concordance of opinions between experts and evaluate the model according to the following parameters: sensitivity (*Se*), specificity (*Sp*), positive predictive value (PPV), negative predictive value (NPV), overall effectiveness (EVG) and the likelihood ratio (LR).

### Bayes model for predicting mortality

The Bayesian prediction model is grounded in a probabilistic framework that integrates prior knowledge with observed data to estimate the likelihood of future events. In the context of predicting mortality associated with EVD, this model facilitates the assessment of risk factors by synthesising available information.

In this study, available data will be collected from experts involved in the clinical management of patients with EVD.

The selected experts were chosen based on their years of experience and involvement in Ebola response efforts. They were drawn from the Ministry of Public Health, Hygiene and Prevention, specifically the Epidemiological Surveillance Division, as well as from the National Biomedical Research Institute, Africa Centres for Disease Control and Prevention (CDC) and the World Health Organization (WHO).

Seven experts participated in this study. With respect to the experts’ opinions, between 3 and 6 experts for a simple model and between 7 and 12 experts for a complex or interdisciplinary model are recommended for Bayesian statistics to ensure diverse opinions. The following steps were taken to develop this mortality model:

#### Expert panel on Ebola case management

To ensure the validity and relevance of our Bayesian prediction model for Ebola mortality, experts were selected based on the following criteria:

- Professional Qualifications: Medical Doctor, Nurse.
- Professional Experience: Minimum of 5 years in managing cases of EVD.
- Research Participation: Contributed to at least two relevant or similar scientific studies.
- Availability: Commitment to active participation throughout all phases of the study, including model development and validation.

Additionally, an extra criterion was introduced:

- Epidemic Response Experience: Active involvement in the Ebola response in Guinea (2014–2016) and in the provinces of North Kivu, South Kivu and Ituri in the DRC (2018–2020), specifically in clinical management activities.

These criteria were established in accordance with recommendations from the WHO and the CDC, emphasising the importance of technical expertise, familiarity with study objectives and effective communication skills in selecting experts for epidemic response missions.

#### Expert interviews and capacity building in Bayesian statistics

The experts selected for the development of the model were interviewed individually. Each presented their curriculum vitae highlighting their experience in the management of the Ebola epidemic. Following this step, the objectives, scientific relevance and methodology of the study were explained, including a simplified overview of the Bayesian approach.

An individual questionnaire was then administered to identify a provisional list of factors associated with EBOV-related mortality. This questionnaire pursued two main objectives, namely to:

- Confirm and retain, among the preselected candidates, those who met the participation criteria.
- Collect preliminary elements necessary for the development of the model.

In practice, each interview was structured around three key components:

- Presentation of the expert’s identity, qualifications, current role and professional experience.
- Briefing on the objectives and methodology of the study.
- Clarification of the operational definitions of the factors associated with EBOV-related mortality in North Kivu, South Kivu and Ituri provinces.

Finally, an official invitation letter, signed by the Dean of the Faculty of Medicine of the University of Kinshasa, was sent to the experts to formalise their participation in the study.

#### Development of a comprehensive list of factors associated with Ebola virus disease severity

The provisional list of factors generated from the expert interviews was used to establish the final list of variables. Experts then proceeded to define the operational parameters for each independent factor. Subsequently, the factors were organised into groups of independent and mutually exclusive variables.

#### Calculation of the prior probability quotient

To determine the prior LR, the following question was posed to obtain the a priori probability (QAPRI) of mortality:

*Among 10 individuals who died from laboratory-confirmed Ebola virus disease, how many exhibited the clinical factors/signs/symptoms included in the grouping?*

To obtain the complementary probability, a second question was asked:

*Among 10 individuals who died from laboratory-confirmed Ebola virus disease, how many did not present the clinical factors/signs/symptoms included in the grouping?*

Based on these questions, the probability of actual death because of EVD (P(D)) for a patient admitted to the ETC with the specified signs or symptoms, as well as the probability of survival (P(S)) given the presence of these signs or symptoms, was determined.

After calculating the arithmetic mean of the different expert estimates, the a priori Ebola mortality prediction ratio was computed using the following formula (Equation 1):


$$Prior\;Likelihood\text{​}Ratio=\frac{P(D)}{P(S)}$$ [Eqn 1]

This approach allowed the integration of expert knowledge with observed clinical patterns to inform a Bayesian predictive model of Ebola mortality.

#### Estimating the likelihood ratio for each factor with respect to fatal outcomes in Ebola virus disease (likelihood ratio)

To estimate the LR for predicting EVD mortality, experts were asked the following question regarding deceased patients:

*Among 10 individuals who died from laboratory-confirmed Ebola virus disease, how many exhibited each of the consensus clinical factors/signs/symptoms: F_1_, F_2_, F_3_, …, F_n_?*

From the experts’ responses, the probability of each factor occurring given death (P(F*_i_*|D)) was calculated (Equation 2):


$$\begin{array}{l} P\left(F_{1}-D\right),P\left(F_{2}-D\right),P\left(F_{3}-D\right),P\left(F_{4}-D\right),P\left(F_{5}-D\right),P\left(F_{6}-D\right),\\ P\left(F_{7}-D\right),P\left(F_{8}-D\right),P\left(F_{9}-D\right),P\left(F_{10}-D\right),P\left(F_{11}-D\right),\\ P\left(F_{12}-D\right),\ldots ,P\left(F_{n}-D\right) \end{array}$$ [Eqn 2]

The complementary probabilities of not having the factors given death were computed as (Equation 3):


$$\begin{array}{l} P\left(\bar{F_{1}}/D\right),P\left(\bar{F_{2}}/D\right),P\left(\bar{F_{3}}/D\right),P\left(\bar{F_{4}}/D\right),P\left(\bar{F_{5}}/D\right),P\left(\bar{F_{6}}/D\right),\\ P\left(\bar{F_{7}}/D\right),P\left(\bar{F_{8}}/D\right),P\left(\bar{F_{9}}/D\right),P\left(\bar{F_{10}}/D\right),P\left(\bar{F_{11}}/D\right),\\ P\left(\bar{F_{12}}/D\right),\ldots ,P\left(\bar{F_{n}}/D\right)=1-P\left(F_{1}-D\right),1-P\left(F_{2}-D\right),\\ 1-P\left(F_{3}-D\right),1-P\left(F_{4}-D\right),1-P\left(F_{5}-D\right),1-P\left(F_{6}-D\right),\\ 1-P\left(F_{7}-D\right),1-P\left(F_{8}-D\right),1-P\left(F_{9}-D\right),1-P\left(F_{10}-D\right),\\ 1-P\left(F_{11}-D\right),1-P\left(F_{12}-D\right),\ldots ,1-P\left(F_{n}-D\right) \end{array}$$ [Eqn 3]

Similarly, to account for survival, experts were asked:

*Among 10 individuals who survived Ebola virus disease, how many exhibited each of the consensus clinical factors/signs/symptoms: F_1_, F_2_, F_3_, …, F_n_?*

This allowed calculation of the probabilities of each factor occurring given survival (P(F*_i_*|S)) (Equation 4):


$$\begin{array}{l} P\left(F_{1}-S\right),P\left(F_{2}-S\right),P\left(F_{3}-S\right),P\left(F_{4}-S\right),P\left(F_{5}-S\right),P\left(F_{6}-S\right),\\ P\left(F_{7}-S\right),P\left(F_{8}-S\right),P\left(F_{9}-S\right),P\left(F_{10}-S\right),P\left(F_{11}-S\right),P\left(F_{12}-S\right),\\ \ldots ,P\left(F_{n}-S\right) \end{array}$$ [Eqn 4]

And the complementary probabilities of not having the factors given survival were derived as (Equation 5):


$$\begin{array}{l} P\left(\bar{F_{1}}/S\right),P\left(\bar{F_{2}}/S\right),P\left(\bar{F_{3}}/S\right),P\left(\bar{F_{4}}/S\right),\\ P\left(\bar{F_{5}}/S\right),P\left(\bar{F_{6}}/S\right),P\left(\bar{F_{7}}/S\right),P\left(\bar{F_{8}}/S\right),\\ P\left(\bar{F_{9}}/S\right),P\left(\bar{F_{10}}/S\right),P\left(\bar{F_{11}}/S\right),P\left(\bar{F_{12}}/S\right),\ldots ,P\left(\bar{F_{n}}/S\right)\\ =1-P\left(F_{1}-S\right),1-P\left(F_{2}-S\right),1-P\left(F_{3}-S\right),1-P\left(F_{4}-S\right),\\ 1-P\left(F_{5}-S\right),1-P\left(F_{6}-S\right),1-P\left(F_{7}-S\right),1-P\left(F_{8}-S\right),\\ 1-P\left(F_{9}-S\right),1-P\left(F_{10}-S\right),P\left(F_{11}-S\right),P\left(F_{12}-S\right),\\ \ldots ,1-P\left(F_{n}-S\right) \end{array}$$ [Eqn 5]

The arithmetic means of the expert estimates were then used to compute the LR for each factor, summarised in a table (see Table 4). The interpretation of the LR values is as follows:

- LR > 1: The factor increases the probability of EVD mortality and has a significant positive impact on predicting death.
- LR < 1: The factor decreases the probability of EVD mortality and has a non-significant or protective effect.
- LR = 0: The factor is neutral; no conclusion can be drawn regarding its effect.

#### Calculation of the posterior probability quotient

Using the QAPRI of mortality and the LRs for each factor, the QAPO for predicting EVD mortality was calculated.

For the prediction of EVD mortality, the QAPO is computed as (Equation 6):


$$QAPO=\frac{P\left(D/F_{1},F_{2},\ldots F_{n}\;ou\;\bar{F_{1}},\bar{F_{2}},\ldots \bar{F_{n}}\right)}{P\left(S/F_{1},F_{2},\ldots F_{n}\;ou\;\bar{F_{1}},\bar{F_{2}},\ldots \bar{F_{n}}\right)}x\frac{P(D)}{P(S)}$$ [Eqn 6]

Equivalently, this can be expressed as the product of individual factor LRs multiplied by the a priori probability ratio (Equation 7):


$$\begin{array}{l} QAPO=\frac{P\left(F_{1}ou\bar{F_{1}}/D\right)}{P\left(F_{1}ou\bar{F_{1}}/S\right)}\text{x}\frac{P\left(F_{2}\;ou\;\bar{F_{2}}/D\right)}{P\left(F_{2}\;ou\;\bar{F_{2}}/S\right)}\\ \text{ x }..\ldots \frac{P\left(F_{n}\;ou\;\bar{F_{n}}/D\right)}{P\left(F_{n}\;ou\;\bar{F_{n}}/S\right)}\text{x}\frac{P(D)}{P(S)} \end{array}$$ [Eqn 7]

Once the QAPO is obtained, the *posterior probability* for a patient to die from EVD is calculated using the following formula (Equation 8):


$$\text{P}=\frac{\text{QAPO}}{1+\text{QAPO}}$$ [Eqn 8]

This approach allows integration of expert knowledge and observed clinical factors to provide a quantitative prediction of mortality for patients with EVD.

### Internal validation of the mortality prediction model

Each expert constructed six hypothetical cases: three in which mortality occurred in the presence of severity factors and three in which mortality occurred in their absence.

We validated the Bayesian predictive model against expert consensus using 42 hypothetical Ebola cases carefully constructed by the experts. Their responses served as the primary reference standard against which model performance was assessed.

Internal validation of the Bayesian model was carried out by comparing the experts’ a priori probability estimates across hypothetical Ebola mortality cases. We then calculated the LR, posterior probability quotients (PPQs) and standard diagnostic performance metrics including *Se, Sp*, PPV, NPV, overall accuracy and assessed inter-rater agreement using Cohen’s kappa statistic.

### Data availability statement

The data available for the development of this predictive model were generated by a group of experts in the clinical management of Ebola during the development of the subjective prognostic model for Ebola patients.

### Ethical considerations

Ethical clearance to conduct this study was obtained from the University of Kinshasa School of Public Health Ethics Committee (No. ESP/CE/114B/2025), dated 12 September 2022. Confidentiality was ensured, as the patients’ names were removed from the database, and analyses were conducted anonymously. Informed consent was obtained from each expert to participate in the development of the subjective Ebola mortality model. They were free to agree or refuse to provide their opinion on the EVD case.

## Results

### Various severity factors listed by the experts in the management of Ebola virus disease

The list of 50 factors (signs and symptoms) indicating the severity of EVD, as cited by the Ebola management experts, is provided in Table 2. These 50 factors are grouped into eight independent factors in Table 2.

### Determination of the a priori probability quotient of Ebola mortality

The experts answered the following question: ‘Out of 10 patients with Ebola, what is the probability of dying for a patient who has various symptoms and/or clinical signs?’ The various answers were used to calculate the QAPRI, which is shown in Table 3^20^ (see Equation 9):


$$QAPRI=\frac{P(D)}{P(S)}=\frac{0.70}{0.30}=2.33$$ [Eqn 9]

**TABLE 3:** A priori probability quotient of mortality.

Expert	1	2	3	4	5	6	7	Sum	*N*	Average
P(D)	0.7	0.7	0.7	0.5	0.7	0.8	0.8	4.9	7	0.70
P(S)	0.3	0.3	0.3	0.5	0.3	0.2	0.2	2.1	7	0.30

The QAPRI value was greater than 1, so the experts’ predictions were that a patient with the EVD factors listed above is more likely to die than to survive.

### Determination of the likelihood ratio according to each factor related to Ebola mortality

Each expert predicted the probability of death in a patient with biologically confirmed EVD according to each individual factor. This prediction was used to determine the LR of each factor for predicting mortality. The results in Table 4 indicate that all eight factors in the LR table have a significantly positive effect on EVD mortality (LR > 1).

**TABLE 4:** Likelihood ratios based on expert responses in terms of the Ebola mortality prediction model.

Probability of dying given factor present or not	Exp1	Exp2	Exp3	Exp4	Exp5	Exp6	Exp7	S	N	M	Probability of surviving given factor present or not	Exp1	Exp2	Exp3	Exp4	Exp5	Exp6	Exp7	S	N	M	LR	Impact
**P (F+/D)**											**P (F +/S)**												
P (F1 +/D)	0.8	0.7	0.8	0.5	1	0.6	0.5	4.9	7	0.70	P (F1 +/S)	0.8	0.1	0.2	0.5	1	0.4	0.3	3.3	7	0.47	1.48	+
P (F2 +/D)	0.9	0.9	0.5	0.7	1	0.6	0.8	5.4	7	0.77	P (F2 +/S)	0.1	0.2	0.5	0.3	0.8	0.4	0.6	2.9	7	0.41	1.86	+
P (F3 +/D)	0.3	0.5	0.8	0.9	0.6	0.8	0.3	4.2	7	0.60	P (F3 +/S)	0.3	0.1	0.2	0.1	0.1	0.2	0.2	1.2	7	0.17	3.50	+
P (F4 +/D)	1	0.8	0.2	0.5	0.9	0.3	0.8	4.5	7	0.64	P (F4 +/S)	0.6	0.2	0.7	0.5	0.8	0.7	0.8	4.3	7	0.61	1.05	+
P (F5 +/D)	0.8	0.7	0.7	0.7	0.8	0.3	0.8	4.8	7	0.69	P (F5 +/S)	0.7	0.2	0.3	0.3	0.6	0.7	0.7	3.5	7	0.50	1.37	+
P (F6 +/D)	0.7	0.5	0.8	0.8	0.2	0.8	0.2	4.0	7	0.57	P (F6 +/S)	0.2	0.1	0.2	0.2	0.0	0.2	0.2	1.1	7	0.16	3.64	+
P (F7 +/D)	0.8	0.8	0.9	0.9	1	0.7	1	6.1	7	0.87	P (F7 +/S)	0.7	0.2	0.1	0.1	0.7	0.3	0.9	3.0	7	0.43	2.03	+
P (F8 +/D)	1	0.7	0.9	0.7	0.7	0.5	1	5.5	7	0.79	P (F8 +/S)	0.4	0.3	0.1	0.3	0.3	0.5	1	2.9	7	0.41	1.90	+
**P (F** −**/D)**											**P (F** −**/S)**												
P (F1 −/D)	0.2	0.3	0.2	0.5	0.0	0.4	0.5	2.1	7	0.30	P (F1 −/S)	0.2	0.9	0.8	0.5	0.0	0.6	0.7	3.7	7	0.53	0.57	−
P (F2 −/D)	0.1	0.1	0.5	0.3	0.0	0.4	0.2	1.6	7	0.23	P (F2 −/S)	0.9	0.8	0.5	0.7	0.2	0.6	0.4	4.1	7	0.59	0.39	−
P (F3 −/D)	0.7	0.5	0.2	0.1	0.4	0.2	0.7	2.8	7	0.40	P (F3 −/S)	0.7	0.9	0.8	0.9	0.9	0.8	0.8	5.8	7	0.83	0.48	−
P (F4 −/D)	0.0	0.2	0.8	0.5	0.1	0.7	0.2	2.5	7	0.36	P (F4 −/S)	0.4	0.8	0.3	0.5	0.2	0.3	0.2	2.7	7	0.39	0.93	−
P (F5 −/D)	0.2	0.3	0.3	0.3	0.2	0.7	0.2	2.2	7	0.31	P (F5 −/S)	0.3	0.8	0.7	0.7	0.4	0.3	0.3	3.5	7	0.50	0.63	−
P (F6 −/D)	0.3	0.5	0.2	0.2	0.8	0.2	0.8	3.0	7	0.43	P (F6 −/S)	0.8	0.9	0.8	0.8	1	0.8	0.8	5.9	7	0.84	0.51	−
P (F7 −/D)	0.2	0.2	0.1	0.1	0.0	0.3	0.0	0.9	7	0.13	P (F7 −/S)	0.3	0.8	0.9	0.9	0.3	0.7	0.1	4.0	7	0.57	0.23	−
P (F8 −/D)	0.0	0.3	0.1	0.3	0.3	0.5	0.0	1.5	7	0.21	P (F8 −/S)	0.6	0.7	0.9	0.7	0.7	0.5	0.0	4.1	7	0.59	0.37	−

Note: LR > 1, the factor increases the probability of dying with EVD; LR < 1, the factor decreases the probability of dying with EVD; LR = 0, the factor is indifferent, so no conclusion can be drawn in this case.

LR, likelihood ratio.

On the basis of the data shown in Table 4, we used the different LRs and a priori probabilities from Table 2 to calculate the corresponding a posteriori probabilities, as shown in Table 5.

**TABLE 5:** Probabilities of mortality as a function of specific combinations of Ebola virus disease severity factors.

Case	EVD risk factors	LR	QAPRI	QAPO	Probability of death from EVD	As % of
1	F1,F2,F3,F4,F5,F6,F7,F8	194.76	2.33	453.8	0.998	99.8
2	F1,F2,F3,F4,F5,F6,F7	37.57	2.33	87.5	0.989	98.9
3	F1,F2,F3,F4,F5,F76,F8	13.55	2.33	31.6	0.969	96.9
4	F1,F2,F3,F4,F5,F7,F8	27.23	2.33	63.5	0.984	98.4
5	F1,F2,F3,F6,F7,F8	78.98	2.33	184.0	0.995	99.5
6	F1,F2,F5,F6,F7,F8	23.77	2.33	55.4	0.982	98.2
7	F1,F4,F5,F6,F7,F8	5.63	2.33	13.1	0.929	92.9
8	F2,F3,F4,F5,F6,F7	14.36	2.33	33.5	0.971	97.1
9	F3,F6,F7	1.22	2.33	2.24	0.740	74.0
10	F1,F2,F4,F5,F8	0.42	2.33	1.0	0.492	49.2
11	F1,F2,F3,F6	1.69	2.33	3.9	0.797	79.7
12	F4,F5,F7	0.06	2.33	0.1	0.119	11.9
13	F1,F2,F6	0.23	2.33	0.5	0.351	35.1
14	F1,F2,F3	0.24	2.33	0.5	0.355	35.5
15	All factors absent	0.00	2.33	0.0	0.006	0.6

EVD, Ebola virus disease; LR, likelihood ratio; QAPRI, priori probability ratio; QAPO, posterior probability ratio.

The table presenting all combinations of Ebola severity factors is provided in Table 1-A1. A total of 248 combinations were identified.

We used the following formulae to determine the PPQ *with all eight mortality-related severity factors present in a patient* (Equation 10):


$$\begin{array}{l} QAPO=\frac{P(F_{1}^{+}/D)}{P(F_{1}^{+}/S)}*\frac{P(F_{2}^{+}/D)}{P(F_{2}^{+}/S)}*\frac{P(F_{3}^{+}/D)}{P(F_{3}^{+}/S)}\\ \;*\frac{P(F_{4}^{+}/D)}{P(F_{4}^{+}/S)}*\frac{P(F_{5}^{+}/D)}{P(F_{5}^{+}/S)}*\frac{P(F_{6}^{+}/D)}{P(F_{6}^{+}/S)}\\ \;*\frac{P(F_{7}^{+}/D)}{P(F_{7}^{+}/S)}*\frac{P(F_{8}^{+}/D)}{P(F_{8}^{+}/S)}*\frac{P(D)}{P(S)}\\ \;QAPO=RV*QAPRI\\ QAPO=1.48*1.86*3.50*1.05*1.37*3.64*2.03*1.90=453.8 \end{array}$$ [Eqn 10]

The same formula was used to determine the PPQ ***with all eight mortality-related severity factors absent in a patient*** (Equation 11):


$$\begin{array}{l} QAPO=\frac{P(F_{1}^{+}/D)}{P(F_{1}^{+}/S)}*\frac{P(F_{2}^{+}/D)}{P(F_{2}^{+}/S)}*\frac{P(F_{3}^{+}/D)}{P(F_{3}^{+}/S)}\\ \;*\frac{P(F_{4}^{+}/D)}{P(F_{4}^{+}/S)}*\frac{P(F_{5}^{+}/D)}{P(F_{5}^{+}/S)}*\frac{P(F_{6}^{+}/D)}{P(F_{6}^{+}/S)}\\ \;*\frac{P(F_{7}^{+}/D)}{P(F_{7}^{+}/S)}*\frac{P(F_{8}^{+}/D)}{P(F_{8}^{+}/S)}*\frac{P(D)}{P(S)}\\ \;QAPO=RV*QAPRI\\ QAPO=0.57*0.39*0.48*0.93*0.63*0.51*0.23*0.37\\ \;*2.33=0.006 \end{array}$$ [Eqn 11]

### Determining the probability of mortality with signs or symptoms related to Ebola virus disease severity

Given the QAPO findings, we calculated the different probabilities of mortality with the different factors (signs and symptoms) related to the severity of EVD using the following formula (Equation 12):


$$P=\frac{QAPO}{1+QAPO}$$ [Eqn 12]

Generally, as shown in this Table 5, when the number of factors increases, the probability of mortality also increases. However, certain factors, even in small numbers, increase the probability of mortality. This is the case for one combination of factors: F3, haemorrhagic syndrome; F6, neurological disorders and F7, biological alteration and dehydration. Moreover, when a patient presents with all eight factors, the chance of survival is close to zero. A total of 248 combinations of predictive factors for Ebola severity were identified. Among these, one combination of eight factors, four combinations of seven factors and two combinations of six factors each yielded mortality predictions ≥ 99.0%. Notably, any combination that included factor F6 was consistently associated with a markedly high probability of mortality (Table 1-A1). The complete set of factor combinations is presented in Table 1-A1.

### Evaluation of the Ebola virus disease mortality prediction model

This model was evaluated by determining the degree of agreement between and among experts in two rounds, depending on the answer to the following question: ‘Out of 6 patients that you know of with different EVD-related factors, how many died?’ The ‘cut-off point’ discrimination criterion was determined to evaluate the effectiveness of the subjective Bayes model.

#### Determination of the discrimination criterion for mortality (cut-off point)

For our study, this criterion was defined as the value at which the model simultaneously exhibited both very high *Se* and *Sp*. The criterion is given by a value between 0.1 and 0.9. Table 6 was constructed for each value between 0.1 and 0.9.

**TABLE 6:** Mortality discrimination criterion – Using hypothetical cases and expert consensus for mortality prediction.

Cut-off point	a	b	c	d	Total	*Se*	*Sp*	PPV	NPV	EVG
0.1	42	0	0	0	42	100.0	#DIV/0!	100.0	#DIV/0!	100.0
0.2	42	0	0	0	42	100.0	#DIV/0!	100.0	#DIV/0!	100.0
0.3	40	2	0	0	42	100.0	0.0	95.2	#DIV/0!	95.2
0.4	38	0	1	3	42	97.4	100.0	100.0	75.0	97.6
0.5	29	0	10	3	42	74.4	100.0	100.0	23.1	76.2
0.6	24	0	8	10	42	75.0	100.0	100.0	55.6	81.0
0.7	20	0	7	15	42	74.1	100.0	100.0	68.2	83.3
0.8	14	1	6	21	42	70.0	95.5	93.3	77.8	83.3
0.9	8	1	5	28	42	61.5	96.6	88.9	84.8	85.7

EVG, overall effectiveness; NPV, negative predictive value; PPV, positive predictive value; *Se*, sensitivity; *Sp*, specificity.

The highest values for both *Se* and *Sp* were obtained at the 0.4 cut-off point, which was chosen for the internal validation of our model, enabling us to compare subjective Bayesian model (SBM) with the expert consensus.

#### Determining the degree of agreement within and between experts

On the basis of the construction of six hypothetical cases by each expert in the first and second rounds and the determination of a consensus on 42 hypothetical cases generated by all the experts, we used the kappa statistic to analyse these results (cut-off point of 0.4).

The results obtained in relation to intraexpert approval showed that six out of seven experts had an acceptable degree of approval for themselves, as their K values were between 0.4 ≤ K ≤ 75. These values are shown diagonally in Table 7. Regarding interexpert approval, 15 out of 21 approvals, which are shown below the diagonal values, were acceptable.

**TABLE 7:** Degree of agreement within and between experts regarding the mortality of Ebola.

Kappa	Intra- and interexpert agreement for EVD mortality						
	E1	E2	E3	E4	E5	E6	E7
E1	0.39	-	-	-	-	-	-
E2	**0.49**	**0.61**	-	-	-	-	-
E3	**0.65**	0.32	**0.49**	-	-	-	-
E4	0.39	**0.65**	0.28	**0.76**	-	-	-
E5	0.33	**0.51**	0.22	**0.67**	**0.85**	-	-
E6	0.39	**0.60**	0.28	**0.62**	**0.57**	**0.76**	-
E7	**0.42**	**0.61**	0.34	**0.55**	**0.54**	**0.50**	**0.90**

Note: The kappa coefficient varies between 0 and 1: < 0.20 slight; 0.21–0.40 medium; 0.41–0.60 moderate; 0.61–0.80 substantial; > 0.80 almost perfect.

EVB, Ebola virus disease.

Given that the majority of experts had an acceptable degree of agreement, we considered the group to be a single person, and we used the arithmetic mean of their estimates to represent agreement to evaluate the Bayes model.

### Internal validation of the mortality prediction model

The internal validation of the mortality prediction model was performed by calculating the following validation parameters: *Se, Sp*, PPV, NPV, overall efficacy value and the kappa statistic. The data used for these different calculations came from the comparison of the subjective Bayes model and the expert consensus on hypothetical cases (Table 8).

**TABLE 8:** Comparison of the subjective Bayes model and expert consensus.

SBM	Mortality from EVD (Hypothetical cases)	Expert consensus (0.4)		Total
		Deaths	Survival	
0.4	Death	38	0	38
	Survival	1	3	4

**Total**	**-**	**39**	**3**	**42**

EVD, Ebola virus disease; SBM, subjective Bayesian model.

Model performance parameter calculations (Equation 13):


$$\begin{array}{l} S_{e}=\frac{a}{a+c}*100=\frac{38}{38+1}*100=97.4\%\\ S_{p}=\frac{d}{b+d}*100=\frac{3}{0+3}*100=100.0\%\\ PPV=\frac{a}{a+b}*100=\frac{38}{38+0}*100=100.\%\\ NPV=\frac{d}{c+d}*100=\frac{3}{1+3}*100=75.0\%\\ Overall\;efficiency\;value=\frac{a+d}{\begin{array}{l} a+b+c+d\\ {}*100=97.6\% \end{array}}*100=\frac{38+3}{38+0+1+3}\\ \begin{array}{llllll} \text{a}1 & 0.90 & & & & \\ \text{a}2 & 0.10 & \text{Po} & 0.98 & \text{MSB }(0.8)\text{ and Expert Consensus} & 0.84\\ \text{b}1 & 0.93 & \text{Pe} & 0.85 & & \\ \text{b}2 & 0.07 & & & & \end{array}\\ P_{0}=\frac{a+d}{a+b+c+d}=\frac{38+3}{38+0+1+3}=0.98\\ P_{e}=\left(a_{1}*b_{1}\right)+\left(a_{2}*b_{2}\right)=0.85\\ K=\frac{P_{0}-P_{e}}{1-P_{e}}=\frac{0.98-0.85}{1-0.85}=0.84 \end{array}$$ [Eqn 13]

The internal validity of the subjective Bayes model for predicting Ebola mortality was established, with a K value of 0.84. The experts’ agreement among themselves on the Ebola severity factors (EVM signs/symptoms) was almost perfect because it was between 0.81 and 1.00.

## Discussion

Knowledge of the factors that worsen the health status of patients with EVD is essential for good medical management.

Our mortality prediction model highlights eight key factors that are considered to worsen the state of health of a patient with EVD. Among these eight factors, the Bayesian model identified three factors (signs and symptoms) as signs of severe EVD. F3 is among these eight factors: haemorrhagic syndrome (unexplained bleeding, bleeding gums, bleeding from the injection, bloody nose, bloody stools, haematemesis, bloody vomit, bloody cough, bleeding from the vagina, bleeding skin, bloody urine and other bleeding). This result aligns with the findings reported by the WHO Ebola response team in West Africa, as well as those of Aylward et al.,^10^ Barry et al.^17^ and Hunt et al.^16^

F6 is also included among the factors indicating severe EVD: neurological disorders (coma, unconsciousness, psychosis, convulsions, aggression and loss of vision). Some researchers have also identified this factor, such as Qin et al.^14^ and Zhang et al.^15^ In addition to F3 and F6, F7 is also included: biological alteration and dehydration (jaundice, altered liver function, altered biological state between diagnosis and follow-up (haematological factors, biochemical factors, protein factors), hyperglycaemia, altered renal function and dehydration). Some researchers have also identified this factor as a sign of severe EVD. This was the case in the study by Schieffelin et al.,^11^ Hunt et al.^16^ and de La Vega et al.^18^

In her article entitled ‘Predicting Ebola Severity: A Clinical Prioritization Score for Ebola virus disease’, Mary-Anne Hartley prioritised clinical signs and symptoms in patients with fatal outcomes because of EVD.^13^ The eight factors identified through the Bayesian approach were consistently validated by the Mary-Anne scoring method, which relies on clinical data. This concordance highlights the robustness of the Bayesian model and underscores the complementarity of the two approaches in predicting EBV severity.

With respect to the Bayesian approach, the results indicate that if a patient exhibits all eight of the factors identified by management experts, the patient has a 99.8% chance of dying; conversely, if all eight factors are absent, the patient has a 0.6% chance of dying.

Among the eight factors listed by the experts, the Bayesian model identified three factors with a very high impact on calculating the probability of death. These factors include F3 (haemorrhagic syndrome), *F6* (neurological disorders) and *F7* (biological alterations and dehydration). The elements of F3, F6 and F7 are shown in Table 5. Apart from these three factors, with an LR well above 1, *F8* (High viral load) and *F2* (alteration of general state and comorbidity) are included, which have an RV close to 2.

When we calculate the probability of a patient dying of Ebola with F3, F6 and F7, the probability of death is 74.0%. However, if we remove F7 and add F1 and F2, that is, combining F1, F2, F3 and F6, the patient will have a 79.7% chance of dying.

According to the scale proposed by Landis and Koch,^20^ given that our K values (0.84) ranged from 0.81 to 1, there was almost perfect agreement between our experts regarding the prediction of mortality as a function of the eight factors listed above.

This study has the limitation of lacking external validation of this Bayesian model using real Ebola death data.

## Conclusion

This study highlights the factors influencing the fatal outcome (mortality) of Ebola patients at ETCs. These factors are of vital importance to those providing medical care to patients in ETCs. When providers know the risk factors for death in advance, management is improved by focusing interventions on the harmful factors that can very easily lead to death. Our results, obtained using Bayes’ mathematical model, are similar to those reported by other researchers using different methodological approaches. Our results are more telling because we considered the opinions of experts and internally validated the model using the kappa statistic.

The results of this research will enable ETCs to save more lives during future Ebola epidemics by effectively triaging patients who require intensive care. This model not only predicts mortality but also supports preparedness by guiding upgrades of ETCs and training experts in advanced clinical care. Such measures could improve patient survival and strengthen outbreak response.

Other contributing factors, for example, economic conditions (availability of medical supplies, therapies, …) at ETCs should be discussed as additional factors that could contribute to a patient’s death despite applying the knowledge from the Bayesian model.

## Appendix 1

**TABLE 1-A1:** Estimated probabilities of mortality based on various combinations of factors associated with the severity of Ebola virus disease.

Number	Different combinations	EVD risk factors	LR	QAPRI	QAPO	Probability of death from EVD	As % of
1	8 factors	F1,F2,F3,F4,F5,F6,F7,F8	194.76	2.33	453.79	0.998	99.8
2	7 factors	F1,F2,F3,F4,F5,F6,F7	37.57	2.33	87.54	0.989	98.9
3		F1,F2,F3,F4,F5,F6,F8	21.55	2.33	50.22	0.980	98.0
4		F1,F2,F3,F4,F5,F7,F8	27.23	2.33	63.45	0.984	98.4
5		F1,F2,F3,F4,F6,F7,F8	89.27	2.33	207.99	0.995	99.5
6		F1,F2,F3,F5,F6,F7,F8	172.32	2.33	401.51	0.998	99.8
7		F1,F2,F4,F5,F6,F7,F8	26.86	2.33	62.59	0.984	98.4
8		F1,F3,F4,F5,F6,F7,F8	40.82	2.33	95.10	0.990	99.0
9		F2,F3,F4,F5,F6,F7,F8	74.45	2.33	173.46	0.994	99.4
10	6 factors	F1,F2,F3,F4,F5,F6	4.16	2.33	9.69	0.906	90.6
11		F1,F2,F3,F4,F5,F7	3.01	2.33	7.02	0.875	87.5
12		F1,F2,F3,F4,F5,F8	3.01	2.33	7.02	0.875	87.5
13		F1,F2,F3,F4,F6,F7	17.22	2.33	40.12	0.976	97.6
14		F1,F2,F3,F4,F6,F8	9.88	2.33	23.02	0.958	95.8
15		F1,F2,F3,F4,F7,F8	12.48	2.33	29.08	0.967	96.7
16		F1,F2,F3,F5,F6,F7	33.24	2.33	77.45	0.987	98.7
17		F1,F2,F3,F5,F6,F8	19.07	2.33	44.43	0.978	97.8
18		F1,F2,F3,F5,F7,F8	24.10	2.33	56.14	0.982	98.2
19		F1,F2,F3,F6,F7,F8	78.98	2.33	184.02	0.995	99.5
20		F1,F2,F4,F5,F6,F7	5.18	2.33	12.07	0.924	92.4
21		F1,F2,F4,F5,F6,F8	2.97	2.33	6.93	0.874	87.4
22		F1,F2,F4,F5,F7,F8	3.76	2.33	8.75	0.897	89.7
23		F1,F2,F4,F6,F7,F8	12.31	2.33	28.69	0.966	96.6
24		F1,F2,F5,F6,F7,F8	23.77	2.33	55.38	0.982	98.2
25		F1,F3,F4,F5,F6,F7	7.87	2.33	18.35	0.948	94.8
26		F1,F3,F4,F5,F6,F8	4.52	2.33	10.52	0.913	91.3
27		F1,F3,F4,F5,F7,F8	5.71	2.33	13.30	0.930	93.0
28		F1,F3,F4,F6,F7,F8	18.71	2.33	43.59	0.978	97.8
29		F1,F3,F5,F6,F7,F8	36.11	2.33	84.15	0.988	98.8
30		F1,F4,F5,F6,F7,F8	5.63	2.33	13.12	0.929	92.9
31		F2,F3,F4,F5,F6,F7	14.36	2.33	33.46	0.971	97.1
32		F2,F3,F4,F5,F6,F8	8.24	2.33	19.19	0.950	95.0
33		F2,F3,F4,F5,F7,F8	10.41	2.33	24.25	0.960	96.0
34		F2,F3,F4,F6,F7,F8	34.12	2.33	79.50	0.988	98.8
35		F2,F3,F5,F6,F7,F8	65.87	2.33	153.47	0.994	99.4
36		F2,F4,F5,F6,F7,F8	10.27	2.33	23.93	0.960	96.0
37		F3,F4,F5,F6,F7,F8	15.60	2.33	36.35	0.973	97.3
38	5 factors	F1,F2,F3,F4,F5	0.58	2.33	1.35	0.575	57.5
39		F1,F2,F3,F4,F6	1.91	2.33	4.44	0.816	81.6
40		F1,F2,F3,F4,F7	2.41	2.33	5.61	0.849	84.9
41		F1,F2,F3,F4,F8	1.38	2.33	3.22	0.763	76.3
42		F1,F2,F3,F5,F6	3.68	2.33	8.57	0.896	89.6
43		F1,F2,F3,F5,F7	4.65	2.33	10.83	0.915	91.5
44		F1,F2,F3,F5,F8	2.67	2.33	6.21	0.861	86.1
45		F1,F2,F3,F6,F7	15.24	2.33	35.50	0.973	97.3
46		F1,F2,F3,F6,F8	8.74	2.33	20.36	0.953	95.3
47		F1,F2,F3,F7,F8	11.04	2.33	25.73	0.963	96.3
48		F1,F2,F4,F5,F6	0.57	2.33	1.34	0.572	57.2
49		F1,F2,F4,F5,F7	0.72	2.33	1.69	0.628	62.8
50		F1,F2,F4,F5,F8	0.42	2.33	0.97	0.492	49.2
51		F1,F2,F4,F6,F7	2.38	2.33	5.53	0.847	84.7
52		F1,F2,F4,F6,F8	1.36	2.33	3.17	0.760	76.0
53		F1,F2,F4,F7,F8	1.72	2.33	4.01	0.800	80.0
54		F1,F2,F5,F6,F7	4.59	2.33	10.68	0.914	91.4
55		F1,F2,F5,F6,F8	2.63	2.33	6.13	0.860	86.0
56		F1,F2,F5,F7,F8	3.32	2.33	7.74	0.886	88.6
57		F1,F2,F6,F7,F8	10.89	2.33	25.38	0.962	96.2
58		F1,F3,F4,F5,F6	0.87	2.33	2.03	0.670	67.0
59		F1,F3,F4,F5,F7	1.10	2.33	2.57	0.720	72.0
60		F1,F3,F4,F5,F8	0.63	2.33	1.47	0.595	59.5
61		F1,F3,F4,F6,F7	3.61	2.33	8.41	0.894	89.4
62		F1,F3,F4,F6,F8	2.07	2.33	4.82	0.828	82.8
63		F1,F3,F4,F7,F8	2.62	2.33	6.10	0.859	85.9
64		F1,F3,F5,F6,F7	6.97	2.33	16.23	0.942	94.2
65		F1,F3,F5,F6,F8	4.00	2.33	9.31	0.903	90.3
66		F1,F3,F5,F7,F8	5.05	2.33	11.77	0.922	92.2
67		F1,F3,F6,F7,F8	16.55	2.33	38.57	0.975	97.5
68		F1,F4,F5,F6,F7	1.09	2.33	2.53	0.717	71.7
69		F1,F4,F5,F6,F8	0.62	2.33	1.45	0.592	59.2
70		F1,F4,F5,F7,F8	0.79	2.33	1.83	0.647	64.7
71		F1,F4,F6,F7,F8	2.58	2.33	6.01	0.857	85.7
72		F1,F5,F6,F7,F8	4.98	2.33	11.61	0.921	92.1
73		F2,F3,F4,F5,F6	1.59	2.33	3.70	0.787	78.7
74		F2,F3,F4,F5,F7	2.01	2.33	4.68	0.824	82.4
75		F2,F3,F4,F5,F8	1.15	2.33	2.68	0.729	72.9
76		F2,F3,F4,F6,F7	6.58	2.33	15.34	0.939	93.9
77		F2,F3,F4,F6,F8	3.78	2.33	8.80	0.898	89.8
78		F2,F3,F4,F7,F8	4.77	2.33	11.12	0.917	91.7
79		F2,F3,F5,F6,F7	12.71	2.33	29.61	0.967	96.7
80		F2,F3,F5,F6,F8	7.29	2.33	16.98	0.944	94.4
81		F2,F3,F5,F7,F8	9.21	2.33	21.46	0.955	95.5
82		F2,F3,F6,F7,F8	30.19	2.33	70.34	0.986	98.6
83		F2,F4,F5,F6,F7	1.98	2.33	4.62	0.822	82.2
84		F2,F4,F5,F6,F8	1.14	2.33	2.65	0.726	72.6
85		F2,F4,F5,F7,F8	1.44	2.33	3.35	0.770	77.0
86		F2,F4,F6,F7,F8	4.71	2.33	10.97	0.916	91.6
87		F2,F5,F6,F7,F8	9.09	2.33	21.17	0.955	95.5
88		F3,F4,F5,F6,F7	3.01	2.33	7.01	0.875	87.5
89		F3,F4,F5,F6,F8	1.73	2.33	4.02	0.801	80.1
90		F3,F4,F5,F7,F8	2.18	2.33	5.08	0.836	83.6
91		F3,F4,F6,F7,F8	4.71	2.33	10.97	0.916	91.6
92		F3,F5,F6,F7,F8	13.80	2.33	32.16	0.970	97.0
93		F4,F5,F6,F7,F8	2.15	2.33	5.01	0.834	83.4
94	4 factors	F1, F2, F3, F4	0.27	2.33	0.62	0.383	38.3
95		F1, F2, F3, F5	0.51	2.33	1.20	0.545	54.5
96		F1, F2, F3, F6	1.69	2.33	3.93	0.797	79.7
97		F1, F2, F3, F7	2.13	2.33	4.96	0.832	83.2
98		F1, F2, F3, F8	1.22	2.33	2.85	0.740	74.0
99		F1, F2, F4, F5	0.08	2.33	0.19	0.157	15.7
100		F1, F2, F4, F6	0.26	2.33	0.61	0.380	38.0
101		F1, F2, F4, F7	0.33	2.33	0.77	0.436	43.6
102		F1, F2, F4, F8	0.19	2.33	0.44	0.307	30.7
103		F1, F2, F5, F6	0.51	2.33	1.18	0.542	54.2
104		F1, F2, F5, F7	0.64	2.33	1.49	0.599	59.9
105		F1, F2, F5, F8	0.37	2.33	0.86	0.461	46.1
106		F1, F2, F6, F7	2.10	2.33	4.90	0.830	83.0
107		F1, F2, F6, F8	1.21	2.33	2.81	0.737	73.7
108		F1, F2, F7, F8	1.52	2.33	3.55	0.780	78.0
109		F1, F3, F4, F5	0.12	2.33	0.28	0.221	22.1
110		F1, F3, F4, F6	0.40	2.33	0.93	0.482	48.2
111		F1, F3, F4, F7	0.50	2.33	1.18	0.540	54.0
112		F1, F3, F4, F8	0.29	2.33	0.67	0.403	40.3
113		F1, F3, F5, F6	0.77	2.33	1.80	0.642	64.2
114		F1, F3, F5, F7	0.97	2.33	2.27	0.694	69.4
115		F1, F3, F5, F8	0.56	2.33	1.30	0.566	56.6
116		F1, F3, F6, F7	3.19	2.33	7.44	0.882	88.2
117		F1, F3, F6, F8	1.83	2.33	4.27	0.810	81.0
118		F1, F3, F7, F8	2.31	2.33	5.39	0.844	84.4
119		F1, F4, F5, F6	0.12	2.33	0.28	0.219	21.9
120		F1, F4, F5, F7	0.15	2.33	0.35	0.261	26.1
121		F1, F4, F5, F8	0.09	2.33	0.20	0.169	16.9
122		F1, F4, F6, F7	0.50	2.33	1.16	0.537	53.7
123		F1, F4, F6, F8	0.29	2.33	0.67	0.400	40.0
124		F1, F4, F7, F8	0.36	2.33	0.84	0.457	45.7
125		F1, F5, F6, F7	0.96	2.33	2.24	0.691	69.1
126		F1, F5, F6, F8	0.55	2.33	1.28	0.562	56.2
127		F1, F5, F7, F8	0.70	2.33	1.62	0.619	61.9
128		F1, F6, F7, F8	2.28	2.33	5.32	0.842	84.2
129		F2, F3, F4, F5	0.22	2.33	0.52	0.341	34.1
130		F2, F3, F4, F6	0.73	2.33	1.70	0.629	62.9
131		F2, F3, F4, F7	0.92	2.33	2.14	0.682	68.2
132		F2, F3, F4, F8	0.53	2.33	1.23	0.552	55.2
133		F2, F3, F5, F6	1.41	2.33	3.28	0.766	76.6
134		F2, F3, F5, F7	1.78	2.33	4.14	0.805	80.5
135		F2, F3, F5, F8	1.02	2.33	2.37	0.704	70.4
136		F2, F3, F6, F7	5.82	2.33	13.57	0.931	93.1
137		F2, F3, F6, F8	3.34	2.33	7.78	0.886	88.6
138		F2, F3, F7, F8	4.22	2.33	9.84	0.908	90.8
139		F2, F4, F5, F6	0.22	2.33	0.51	0.338	33.8
140		F2, F4, F5, F7	0.28	2.33	0.65	0.392	39.2
141		F2, F4, F5, F8	0.16	2.33	0.37	0.270	27.0
142		F2, F4, F6, F7	0.91	2.33	2.12	0.679	67.9
143		F2, F4, F6, F8	0.52	2.33	1.21	0.548	54.8
144		F2, F4, F7, F8	0.66	2.33	1.53	0.605	60.5
145		F2, F5, F6, F7	1.75	2.33	4.08	0.803	80.3
146		F2, F5, F6, F8	1.01	2.33	2.34	0.701	70.1
147		F2, F5, F7, F8	1.27	2.33	2.96	0.747	74.7
148		F2, F6, F7, F8	4.16	2.33	9.70	0.907	90.7
149		F3, F4, F5, F6	0.33	2.33	0.78	0.437	43.7
150		F3, F4, F5, F7	0.42	2.33	0.98	0.495	49.5
151		F3, F4, F5, F8	0.24	2.33	0.56	0.360	36.0
152		F3, F4, F6, F7	1.38	2.33	3.21	0.763	76.3
153		F3, F4, F6, F8	0.79	2.33	1.84	0.648	64.8
154		F3, F4, F7, F8	1.00	2.33	2.33	0.700	70.0
155		F3, F5, F6, F7	2.66	2.33	6.20	0.861	86.1
156		F3, F5, F6, F8	1.53	2.33	3.56	0.781	78.1
157		F3, F5, F7, F8	1.93	2.33	4.50	0.818	81.8
158		F3, F6, F7, F8	6.33	2.33	14.74	0.936	93.6
159		F4, F5, F6, F7	0.42	2.33	0.97	0.492	49.2
160		F4, F5, F6, F8	0.24	2.33	0.55	0.357	35.7
161		F4, F5, F7, F8	0.30	2.33	0.70	0.412	41.2
162		F4, F6, F7, F8	0.99	2.33	2.30	0.697	69.7
163		F5, F6, F7, F8	0.27	2.33	0.62	0.383	38.3
164	3 factors	F1, F2, F3	0.24	2.33	0.55	0.355	35.5
165		F1, F2, F4	0.04	2.33	0.09	0.079	7.9
166		F1, F2, F5	0.07	2.33	0.17	0.142	14.2
167		F1, F2, F6	0.23	2.33	0.54	0.351	35.1
168		F1, F2, F7	0.29	2.33	0.68	0.406	40.6
169		F1, F2, F8	0.17	2.33	0.39	0.282	28.2
170		F1, F3, F4	0.06	2.33	0.13	0.115	11.5
171		F1, F3, F5	0.11	2.33	0.25	0.201	20.1
172		F1, F3, F6	0.26	2.33	0.60	0.374	37.4
173		F1, F3, F7	0.45	2.33	1.04	0.510	51.0
174		F1, F3, F8	0.26	2.33	0.60	0.374	37.4
175		F1, F4, F5	0.02	2.33	0.04	0.038	3.8
176		F1, F4, F6	0.06	2.33	0.13	0.114	11.4
177		F1, F4, F7	0.07	2.33	0.16	0.140	14.0
178		F1, F4, F8	0.04	2.33	0.09	0.085	8.5
179		F1, F5, F6	0.11	2.33	0.25	0.199	19.9
180		F1, F5, F7	0.13	2.33	0.31	0.238	23.8
181		F1, F5, F8	0.08	2.33	0.18	0.152	15.2
182		F1, F6, F7	0.44	2.33	1.03	0.506	50.6
183		F1, F6, F8	0.25	2.33	0.59	0.371	37.1
184		F1, F7, F8	0.32	2.33	0.74	0.427	42.7
185		F2, F3, F4	0.10	2.33	0.24	0.192	19.2
186		F2, F3, F5	0.20	2.33	0.46	0.314	31.4
187		F2, F3, F6	0.64	2.33	1.50	0.600	60.0
188		F2, F3, F7	0.81	2.33	1.90	0.655	65.5
189		F2, F3, F8	0.47	2.33	1.09	0.521	52.1
190		F2, F4, F5	0.03	2.33	0.07	0.067	6.7
191		F2, F4, F6	0.10	2.33	0.23	0.190	19.0
192		F2, F4, F7	0.13	2.33	0.30	0.228	22.8
193		F2, F4, F8	0.07	2.33	0.17	0.145	14.5
194		F2, F5, F6	0.19	2.33	0.45	0.311	31.1
195		F2, F5, F7	0.25	2.33	0.57	0.363	36.3
196		F2, F5, F8	0.14	2.33	0.33	0.247	24.7
197		F2, F6, F7	0.80	2.33	1.87	0.652	65.2
198		F2, F6, F8	0.46	2.33	1.07	0.518	51.8
199		F2, F7, F8	0.58	2.33	1.36	0.576	57.6
200		F3, F4, F5	0.05	2.33	0.11	0.098	9.8
201		F3, F4, F6	0.15	2.33	0.36	0.262	26.2
202		F3, F4, F7	0.19	2.33	0.45	0.310	31.0
203		F3, F4, F8	0.11	2.33	0.26	0.205	20.5
204		F3, F5, F6	0.29	2.33	0.69	0.407	40.7
205		F3, F5, F7	0.37	2.33	0.87	0.465	46.5
206		F3, F5, F8	0.21	2.33	0.50	0.332	33.2
207		F3, F6, F7	1.22	2.33	2.84	0.740	74.0
208		F3, F6, F8	0.70	2.33	1.63	0.620	62.0
209		F3, F7, F8	0.88	2.33	2.06	0.673	67.3
210		F4, F5, F6	0.05	2.33	0.11	0.097	9.7
211		F4, F5, F7	0.06	2.33	0.14	0.119	11.9
212		F4, F5, F8	0.03	2.33	0.08	0.072	7.2
213		F4, F6, F7	0.19	2.33	0.44	0.307	30.7
214		F4, F6, F8	0.11	2.33	0.25	0.203	20.3
215		F4, F7, F8	0.14	2.33	0.32	0.243	24.3
216		F5, F6, F7	0.37	2.33	0.86	0.461	46.1
217		F5, F6, F8	0.21	2.33	0.49	0.329	32.9
218		F5, F7, F8	0.05	2.33	0.12	0.107	10.7
219		F6, F7, F8	0.87	2.33	2.03	0.670	67.0
220	2 factors	F1, F2	0.03	2.33	0.08	0.070	7.0
221		F1, F3	0.05	2.33	0.12	0.103	10.3
222		F1, F4	0.01	2.33	0.02	0.018	1.8
223		F1, F5	0.01	2.33	0.03	0.033	3.3
224		F1, F6	0.05	2.33	0.11	0.102	10.2
225		F1, F7	0.06	2.33	0.14	0.125	12.5
226		F1, F8	0.04	2.33	0.08	0.076	7.6
227		F2, F3	0.09	2.33	0.21	0.174	17.4
228		F2, F4	0.01	2.33	0.03	0.032	3.2
229		F2, F5	0.03	2.33	0.06	0.059	5.9
230		F2, F6	0.09	2.33	0.21	0.172	17.2
231		F2, F7	0.11	2.33	0.26	0.207	20.7
232		F2, F8	0.06	2.33	0.15	0.131	13.1
233		F3, F4	0.02	2.33	0.05	0.047	4.7
234		F3, F5	0.04	2.33	0.10	0.088	8.8
235		F3, F6	0.14	2.33	0.31	0.239	23.9
236		F3, F7	0.17	2.33	0.40	0.285	28.5
237		F3, F8	0.10	2.33	0.23	0.186	18.6
238		F4, F5	0.01	2.33	0.01	0.015	1.5
239		F4, F6	0.02	2.33	0.05	0.047	4.7
240		F4, F7	0.03	2.33	0.06	0.058	5.8
241		F4, F8	0.02	2.33	0.04	0.034	3.4
242		F5, F6	0.04	2.33	0.09	0.087	8.7
243		F5, F7	0.05	2.33	0.12	0.107	10.7
244		F5, F8	0.03	2.33	0.07	0.064	6.4
245		F6, F7	0.17	2.33	0.39	0.282	28.2
246		F6, F8	0.10	2.33	0.23	0.184	18.4
247		F7, F8	0.12	2.33	0.28	0.221	22.1
248	Absence of factors	0 factors	0.003	2.33	0.01	0.006	0.6

EVD, Ebola virus disease; LR, likelihood radio; QAPRI, priori probability ratio; QAPO, posterior probability ratio.
